# A Multi-User Collaborative AR System for Industrial Applications

**DOI:** 10.3390/s22041319

**Published:** 2022-02-09

**Authors:** Junyi Wang, Yue Qi

**Affiliations:** 1 State Key Laboratory of Virtual Reality Technology and Systems, School of Computer Science and Engineering, Beihang University, Beijing 100191, China; wangjy0524@163.com; 2 Peng Cheng Laboratory, Shenzhen 518067, China; 3 Qingdao Research Institute of Beihang University, Qingdao 266104, China

**Keywords:** augmented reality system, multi-user collaborative system, collaborative localization, industrial applications

## Abstract

Augmented reality (AR) applications are increasingly being used in various fields (e.g., design, maintenance, assembly, repair, training, etc.), as AR techniques help improve efficiency and reduce costs. Moreover, collaborative AR systems extend applicability, allowing for collaborative environments for different roles. In this paper, we propose a multi-user collaborative AR system (aptly called the “multi-user collaborative system”, or MUCSys); it is composed of three ends—MUCStudio, MUCView, and MUCServer. MUCStudio aims to construct industrial content with CAD model transformation, simplification, database update, marker design, scene editing, and exportation, while MUCView contains sensor data analysis, real-time localization, scene loading, annotation editing, and virtual–real rendering. MUCServer—as the bridge between MUCStudio and MUCView—presents collaborative and database services. To achieve this, we implemented the algorithms of local map establishment, global map registration, optimization, and network synchronization. The system provides AR services for diverse industrial processes via three collaborative ways—remote support, collaborative annotation, and editing. According to the system, applications for cutting machines were presented to improve efficiency and reduce costs, covering cutting head designs, production line sales, and cutting machine inspections. Finally, a user study was performed to prove the usage experience of the system.

## 1. Introduction

Augmented reality (AR) provides a view composed of the real physical world and digital virtual elements, covering a set of technologies, such as localization, rendering, scene understanding, etc. In recent years, AR has trended in various areas, including in the entertainment and medical fields [1,2,3,4,5,6,7,8].

Limitations in devices and algorithms have constrained AR applications up until a few years ago. Nowadays, there is no lack of devices, due to the popularization of mobile devices, such as smartphones and tablets, as well as different types of AR glasses. Moreover, due to technological developments, we have achieved accurate localization and map establishments in general scenes, resulting in stable virtual–real fusion performances. Release libraries, such as ARCore and ARKit, contribute to a variety of applications, further lowering the development threshold.

Pertaining to current research in the AR field—AR industrial systems are mainly applied toward design, maintenance, assembly, repair, training, and inspection processes, with the goal of improving performance and reducing costs and machine losses [9,10]. In the design stage, two application scenarios are the CAD product model and factory layout [11,12,13,14]. Regarding CAD, with AR techniques and mobile devices, the designer can visualize a 3D virtual target object directly superimposed on the real environment. Moreover, with sensor data, the motion of the virtual model is simulated. Based on virtual–real fusion, designers can modify the shape of the CAD model. In the factory layout, the complex manufacturing system always comprises various parts, such as robots, automated guided vehicles, pallet changers, conveyors, etc. To solve this problem, AR systems can arrange these elements in the actual factory, providing the visualization for making decisions.

Similar to the above request, the inspection process can be enhanced by AR technology. Industrial product creation is a complex procedure, it usually includes conception, design, and realization. It is necessary to check whether errors occur after product realization. To achieve this goal, the inspection procedure will involve an organized examination of a particular device. AR is regarded as a promising technology to set up the inspection process [15,16,17,18]. Another inspection application involves the patrol inspection of the running machine. With Internet of Things (IoT) support, the inspector can obtain real-time running information to judge the status of the target machine.

Other AR research fields cover maintenance, repair, and assembly, with the goal of reducing time, costs, and the error rate [19,20,21]. Operators would need to conduct continuous attention switches between the manual and device during some complex tasks, which may involve a high cognitive load. AR technology focuses on this mission by overlaying virtual information onto the real part, to help operators complete their tasks. Virtual information (commonly) comprehends 3D model animations, instruction audio, and textual labels aligned to the machines, to provide detailed operations of the next steps [22]. Moreover, AR applications can enable remote technicians to interactively support maintainers when AR aids are not sufficient. Training—closely associated with the maintenance, repair, and assembly tasks—is another application direction in the industry domain [23,24,25]. Instructors, teachers, and trainers tend to explore new methods to enhance learning efficiency and experience. AR techniques meet these demands and can present novel experiences in the learning process. Compared with traditional approaches, on the one hand, multimedia information enhances user interactions and increases users’ interests. On the other hand, various perilous or equipment-worn procedures could also be directly simulated by AR systems.

**Figure 1:**
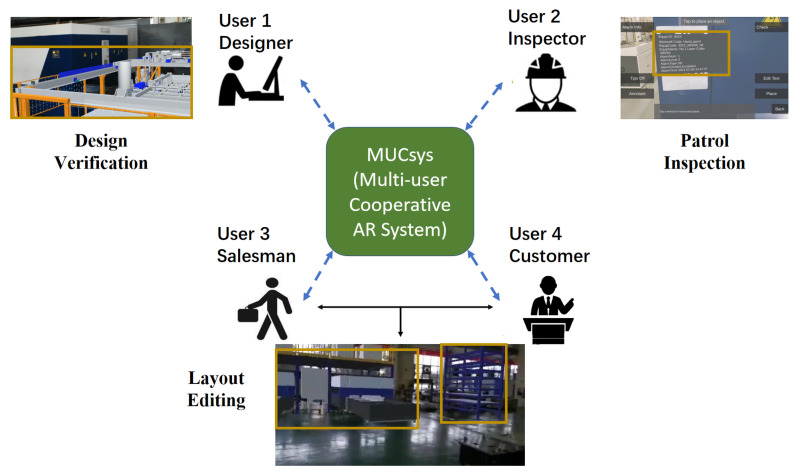
A collaborative AR system for multiple users, including designers, inspectors, salesmen, and customers. In each figure, the virtual model is marked with a brown box.

In addition to the above, collaborative AR systems further satisfy industrial demands by providing cooperation among operators [26,27,28]. In summary, as shown in Figure 1, we propose a multi-user collaborative AR system, called MUCSys, for industrial applications.

Compared to previous AR systems, our MUCSys has the following peculiarities: (1) the developed system can support various industrial processes, such as design, inspection, assembly, sales, etc.; (2) three collaborative AR modes are presented to bridge interactions among different roles. In detail, remote support provides remote assistance between experts and workers (e.g., salesmen, maintenance people, or after-sales staff) in actual scenes. Collaborative annotation offers asynchronous adding, deleting, or modifying operations for different workers. Meanwhile, multiple users can collectively complete target tasks (e.g., production layout) via collaborative editing in real-time.

The whole system is composed of MUCStudio in PC, and MUCServer and MUCView in mobile devices. In detail, MUCStudio aims for industrial scene construction, while MUCView mainly provides AR experiences for users. Moreover, interlinking MUCStudio and MUCView, MUCServer presents database and collaborative services. To prove the effectiveness of the proposed system, laser cutting machine applications containing designs, sales, and inspections are provided.

In summary, compared to prior works, our work provides the following contributions.

1. We propose a multi-user collaborative AR system for laser cutting machines, containing three collaborative modes—remote support, annotation, and editing. The system comprises MUCStudio for scene generation, MUCView for AR experience, and MUCServer for collaboration.
2. To implement collaborative services, we designed several algorithms. Firstly, the local map was established with additional marker and QR code inputs based on ORB-SLAM3. Secondly, we propose a map registration pipeline by combining ORB match and point cloud registration. Global optimization was performed to promote the relative transformation accuracy. Finally, the network synchronization was exploited to improve the user experience.
3. Based on the system, three applications for laser cutting machines were developed—cutting head design, production line sale, and cutting machine inspection.
4. A user study was conducted to demonstrate the user experience of the system.

The remainder of the paper is organized as follows: related works are summarized in Section 2. Section 3 illustrates the detailed system composition and implementation. Based on the system, Section 4 presents three application scenarios involving laser cutting machines—designs, sales, and inspections. Section 5 presents the results of the user study and Section 6 presents the conclusions.

## 2. Related Work

### 2.1. Design

In the design process, AR systems can identify and avoid design errors in the early stages to reduce time, costs, and the number of physical prototypes. The CAD product model and factory layout design are two common application scenarios.

In the context of the CAD product model, Jimeno et al. [11] implemented an AR system with low-cost computational elements that allowed customers to check the quality of the footwear model from an aesthetic perspective. Georgel et al. [29] developed zoom-and-pan tools within mixed views to solve undocumented discrepancies between the CAD model and the final object. Caruso et al. [30], aiming for interactions with virtual objects superposed in a real environment, developed an interactive AR system that integrates stereoscopic visualization and fog screen display technology. Mourtzis et al. [13], to support the customer integration in the design stage, presented a framework consisting of a network design tool with a smart search algorithm, and a mobile application based on AR technology. The BIM-AR system [27], by implementing marker-based AR, provides the ability to view, interact, and collaborate with 3D and 2D BIM data.

Besides the CAD product model, planning a suitable factory layout is also a challenge. Shariatzadeh et al. [14], focusing on rapid factory design and planning, and based on actual industrial demands, took different layouts into consideration to determine the main functionalities. Pentenrieder et al. [31] presented a complete system composed of the requirement analysis, developing process and realization. Based on the system, a concrete usage example for factory planning was declared. Kokkas et al. [12] performed layout planning of machinery with AR tools, aiming to evaluate the suggested layouts through non-measurable factors.

In the laser cutting machine applications of our system, both the CAD product model and layout design can be verified through SMCStudio and SMCView. Through SMCStudio, the user designs the production line layout or the model position relative to the real environment by the marker. Then, SMCView superposes the virtual model on the real environment. Moreover, multiple users can cooperate through the SMCServer. With sensor data supporting, SMCView also simulates the trajectory and drives the virtual part to provide more visualization information for designers.

### 2.2. Maintenance, Assembly, Repair, and Training

In maintenance, repair, assembly, and training processes, AR technology can efficiently help operators understand the procedure of the tasks by means of overlaying the virtual instruction information onto the real environment [24,32]. The AR-based manual has shown that it can reduce the time and costs, and improve performance.

An early attempt to support technicians in simple maintenance procedures involved the intent-based illustration system proposed by Feiner et al. [33]. Then, various AR applications in manufacturing activities, such as maintenance, repair, and assembly were developed and demonstrated [19,21,34]. The project STAR-MATE [35] realized one of the earliest multi-modal interactions with a virtual pointing device and voice-based commands. In other projects, researchers provided mobile AR experiences in performing procedures in an industrial environment (instead of staying at a stationary workbench) [31,36,37]. Hou et al. [20] configured a prototype animated AR system for assembly tasks that are commonly instructed by text documentation. Then, a series of experiments were conducted to prove the effectiveness of the system. Chen et al. [26] developed a location aware AR collaborative framework for FMM with the interaction between users and facilities.

AR-based industrial training tasks are always related to the repair, assembly, and maintenance processes, with the aim of improving learning efficiency and reducing device costs [24]. Various industrial domains used AR technologies in training and supporting tasks, such as aerospace [23,38], automotive [39], and industrial plants [40]. A flexible training framework for maintenance was proposed by Sanna et al. [25] to address the problem of creating AR content [41], which allowed instructors to generate maintenance procedures conveniently. Wang et al. [28] described a remote collaborative for training in the manufacturing industry, enabling a remote expert to train a local worker in a physical assembly task.

Through our MUCSys, it is convenient to construct maintenance, assembly, repair, and training scenarios. However, in our applications for cutting machines, these kinds of demands are slight, as workers receive meticulous training and need to pass an examination before entering the workplace. Therefore, applications about maintenance, assembly, repair, and training are not provided.

### 2.3. Inspection

After the design and production phases, the product is realized. On account of the whole process complexity, it is essential to check whether errors and differences occurred during the phases, which can be achieved with AR technology [16].

An AR-based reconfigurable framework for inspection was proposed by Ramakrishna et al. [17], prioritizing the checklist by detecting the parts with deep learning. The framework can be utilized in various applications, such as industrial maintenance, the health sector, and so on. Wasenmuller et al. [18], regarding the discrepancy check task in industrial AR, presented a new approach that consisted of two-step depth mapping, semi-automatic alignment, and a 3D discrepancy check. The approach showed the superior performance compared with the state-of-the-art 3D discrepancy check. Munoz et al. [15] proposed a novel AR-based user interface for quality control inspection of car body surface production lines, to reduce working stress and lift the ergonomics of workers.

The inspection pipeline introduced above mainly focuses on realizing inspections to ensure quality. In our paper, after analyzing the requirements of the laser cutting machine, we employed our system to the patrol inspection process, to judge if the device was under the normal operating conditions and to find the running problem in the early stage.

## 3. System Design

### 3.1. Overall Structure

**Figure 2:**
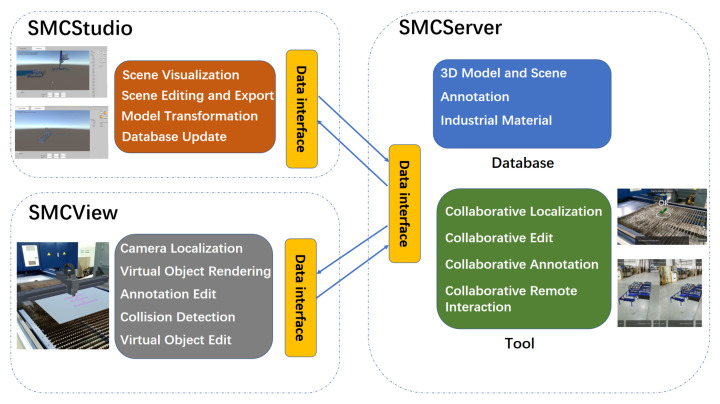
Outline architecture of MUCSys.

As demonstrated in Figure 2, the whole system is composed of three parts, covering MUCServer, MUCStudio, and MUCView. In detail, the MUCServer mainly provides a database service and multi-user collaboration. Correspondingly, MUCStudio runs on a PC and focuses on database management and industrial content production. Users can update the database that contains 3D industrial models, materials, and user annotations by MUCStudio. Moreover, MUCStudio aims to construct an industrial scene for designs, sales, or other applications. The functions include model transformation, visualization, pose editing, marker design, and exportation. MUCView—accepting the exported scene by MUCStudio—renders virtual models superposed onto the real environment by AR technology. When multiple users perform collaborative works, MUCView provides three collaborative ways from the MUCServer to support cooperation among different workers.

### 3.2. MUCStudio

MUCStudio looks at “constructing” the industrial scene for the AR experience. On the one hand, an industrial scene always covers various products with different material warehouses, marking machines, transmissions, etc. Through MUCStudio, users can select their wanted products to generate the final scene. On the other hand, the designed virtual scene should be appropriately fused with the real environment. MUCStudio adopted the marker to solve this problem. By designing markers in virtual and real scenes, both coordinate systems are unified, achieving an ideal virtual–real fusion effect.

The interface of MUCStudio is illustrated in Figure 2, covering CAD model processing, database establishment, and scene creation. In detail, the CAD operation module implements a CAD format transformation and simplification. After the process, the lightweight model with common 3D formats (sty, obj, ply, etc.) is obtained to be visualized in MUCStudio. In the actual implementation, we exploited CAD Exchanger SDK to accomplish the task, which is a free library to develop fast and robust 3D applications. According to the demand, users can establish their model database in MUCServer. While creating the scene, users download the arbitrary model from the built database. With the model database, designers can construct their wanted scene to verify the CAD model. For each model, MUCStudio provides the editing function of its position, orientation, and scale, as shown at the left of Figure 2. Furthermore, marker creation and editing help users ensure the relative poses between the real environment and virtual models.

### 3.3. MUCView

The purpose of MUCView is to provide AR experiences for users. It is mainly composed of camera tracking, collision detection, object editing, data loader, annotation editing, and virtual object rendering.

The camera tracking and object editing parts are implemented by Google ARCore, enabling the mobile device to understand and track its position relative to the world. Based on the obtained feature points and camera pose, the virtual object is rendered in the real environment by Unity. During the collaborative mode, the pose of the virtual object is then transformed by a relative matrix from MUCServer. After rendering the virtual object, users can edit its position, orientation, and scale. For the convenience of moving objects, MUCView presents a grouping function to bind different models. Moreover, to void collision detection during layout design, MUCView gives a reminder when virtual objects have collisions. After determining the model position, the data loader module loads sensor data to drive the model to perform the motion. Annotation editing offers the user addition, modification, and deletion of the virtual annotation, which will be illustrated in the next subsection on patrol inspection.

Through the communication with MUCServer, MUCView achieves multiple collaborative operations covering remote support, collaborative editing, and annotation.

### 3.4. MUCServer

**Figure 3:**
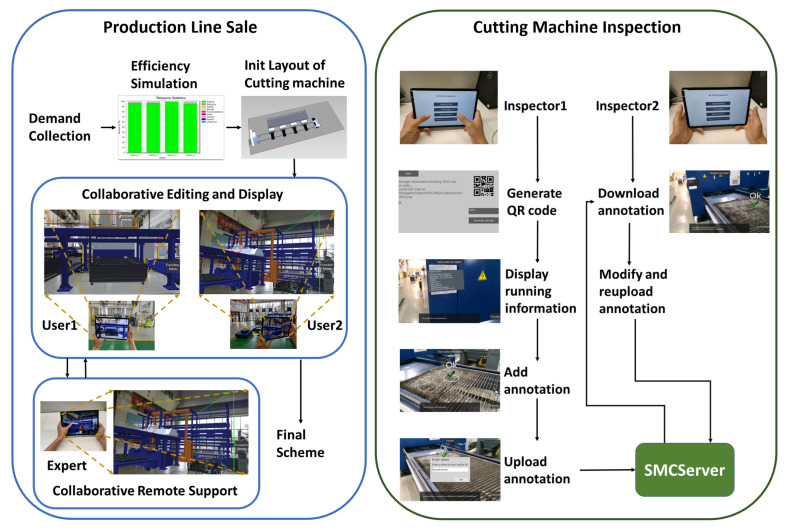
Application process in production line sale and running cutting machine inspection.

The MUCServer mainly performs database and collaborative services. The database covers CAD, 3D model, annotation, and scene information that can be updated by MUCStudio and MUCView. The collaborative service provides three ways to satisfy different demands. When users need the help of remote experts, remote support presents virtual–real video transmissions and shared interactions, demonstrated in Figure 3. The expert receives the AR video from the user and gives the guide to the AR video in real-time. Moreover, collaborative editing offers a cooperative environment for multiple users. Using layout adjustment as an example, after dividing the layout work, each user concentrates on his own part, which can improve efficiency. Collaborative annotation—different from the above—is not real-time. The annotation information is bound to the machine. Different operators can achieve cooperation through checking, adding, modifying, or deleting the annotation.

**Figure 4:**
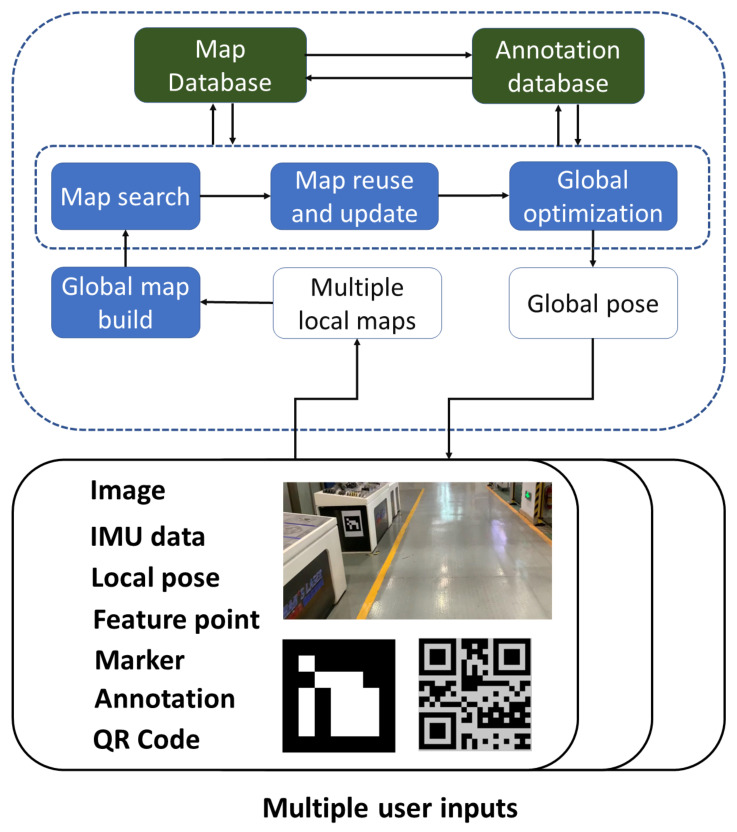
The framework for collaborative remote supporting, editing, and annotation.

In the implementation, remote support technology relies on the video transmission realized by the FFmpeg library, while collaborative editing and annotation are based on the collaborative localization and map establishment among different devices illustrated in Figure 4. The whole pipeline consists of local map establishment, global map building, search, reuse, update, and global optimization. Based on map registration, we fused local maps to establish the global map. Then we searched the map database to perform map reuse and update. Meanwhile, because of connections between map and annotation, the previous annotations were also obtained. Finally, the evaluated pose and annotations were passed to the MUCView.

**Local map.** With the gathered image, IMU data, local camera pose by ARCore, feature point, detected marker, QR code, and annotation, the local map is established by ORB-SLAM3 [42], covering sparse point cloud and keyframe dataset. In particular, there are many similar views in the industrial scene, increasing the difficulty of collaborative localization. To solve this problem, we added QR codes and markers into the image match process and gave them more confidence. Similarly, in local optimization, feature points in QR codes and markers are also taken into consideration. In the actual implementation, each machine connected to IoT is always pasted with a QR code, so that user can obtain running information. The markers are always placed in spaces with less textures and corners, which contributes to localization and mapping.

**Map registration.** To unify the coordinate system among different local maps, we needed to calculate the relative transformation matrices. Global map building, search, reuse, and update are all based on the pipeline. The map registration firstly performs the ORB feature match among keyframes from different local maps. As with ORB-SLAM3, when enough feature correspondences were built, we then exploited global optimization to determine the final relative pose. Because of the device difference and changes of environments, the ORB feature match may fail. In this situation, we leveraged the point cloud registration algorithm instead.

In the registration pipeline, the key points with significant intrinsic shape characteristics were first extracted to reduce subsequent calculations. Then, the FPFH method was used to describe the characteristics of these points. By correspondences between two point clouds, the initial registered matrix was calculated to reduce the range of randomly selected interior points in RANSAC. Meanwhile, the pre-rejection method based on geometric constraints was also exploited to accelerate the coarse registration process. Finally, by using the approximate nearest neighbor ICP method, the fine registration matrix was obtained for map alignment.

**Global optimization.** A variety of SLAM systems utilize the BA process to reduce accumulative errors [42,43]. Multiple components covering camera poses and map points are jointly optimized by taking advantage of the traditional BA pipeline. In our application scenarios, feature points in makers and QR codes are considered with larger weights. Now, we provide the detailed formula of the whole BA. For convenience, feature points, camera poses, and marker points are denoted as $P=P_{i}$, $C=C_{i}$, $M=M_{i}$∑ respectively. Then our BA is expressed as the following nonlinear optimization problem,

(1) $$\begin{array}{c} C^{*},P^{*},O^{*}=argmin\sum_{C_{i},P_{i},M_{i}}\left(∥E\left(P_{i},C_{i}\right)∥+\alpha \;*∥E\left(M_{i},C_{i}\right)∥\right), \end{array}$$

where $E(P_{i},C_{i})$, $E(M_{i},C_{i})$ denote the camera-point and camera-maker errors. Both errors are denoted by the 3D point projection error in ORB SLAM3 [42] with the following form,

(2) $$\begin{array}{cc} \; & E\left(P_{i},C_{i}\right)=\pi \left(R_{c}P_{i}\right)-d, \end{array}$$

(3) $$\begin{array}{cc} \; & E\left(M_{i},C_{i}\right)=\pi \left(R_{c}M_{i}\right)-d_{m}, \end{array}$$

where $R_{c}$ is the transformation matrix from world space to camera space, π represent the matrix from camera space to pixel space, **$d,d_{m}$** denote pixel coordinates of 3D points.

**Network synchronization.** Besides the collaborative localization, the network synchronization method is also important in promoting the user experience. Based on the adaptive lockstep algorithm, we set an upper threshold for the synchronization interval on the server to prevent clients with poor networks from delaying other clients. In the network environment of each client, the synchronization interval was calculated in real-time, instead of using a fixed-length synchronization interval. This was to avoid further deterioration of network conditions caused by short intervals and to prevent too long intervals from making clients with good network conditions unable to obtain sufficient fluency. In addition, smooth interpolation of the local client, optimization of local user operation delay, and server lag compensation were also performed.

## 4. Applications in Cutting Machine

In this section, three applications are presented (by exploiting our proposed system, containing cutting head design, production line sale, and cutting machine inspection). The design process demonstrates the CAD model verification procedure by MUCStudio and MUCView. Sales and inspection depend on AR collaboration. The remote support and collaborative editing establish an environment for salesmen, customers, and experts, while a collaborative annotation provides cooperation among different inspectors.

### 4.1. Design

**Figure 5:**
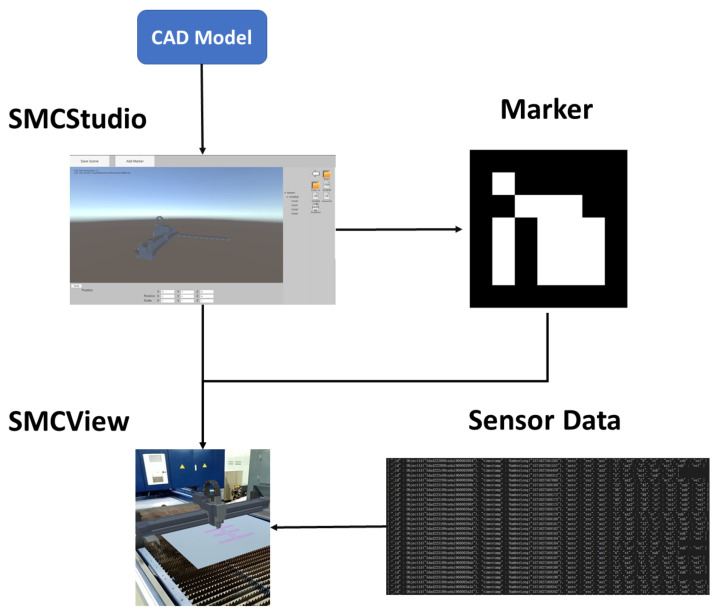
Application process in cutting head design. Through MUCStudio, we added a marker and determined the relative pose between the cutting head and marker. Then we adopted MUCView to drive the cutting head to move with the designed trajectory after loading the scene and sensor data.

The usage in the design stage involves verifying the CAD model through virtual–real fusion and a data-driven simulation. As stated in Figure 5, using cutting head design as an example, after completing one version of the CAD model, the system can help discover the design problem through visualizing the 3D model and driving the model move by sensor data.

To achieve the goal, the designer employs MUCStudio to transform the cutting head CAD model to “obj” format for visualization. Then the relative position between the cutting head and marker is determined. Furthermore, the sensor data are bound to the cutting head model. Then the designer prints the marker and pastes it to the corresponding position as our placing in MUCStudio. Finally, to verify the design of the model, MUCView is exploited to render the virtual CAD model onto the real machine.

### 4.2. Sale

The demand for laser cutting machine sales is at the layout of the production line. In the actual sale process, based on the cutting demand and field condition of the customer, a salesman should determine the cutting machine type, its corresponding automatic feeder, marking machine, and device information. However, due to differences in the actual field, the selection and layout of the production line are time-consuming. Moreover, no visualization of the production line increases communication difficulty between the salesmen and customers.

The sales process is illustrated in Figure 3, by utilizing our system. Firstly, the salesman gathers user demand that contains field size, cutting materials, capacity, etc. To decide the cutting machine type, it is essential to analyze cutting efficiency and requirement. After ensuring the production line composition, MUCView generates the initial layout of the whole production line. Then, in the actual field, the salesman and customer exploit collaborative layout editing to determine the layout. In particular, the salesman can also apply for remote collaborative assistant by the expert when encountering a difficult case. The remote expert receives a virtual–real fusion video and makes simple marks to communicate with the salesman in real-time.

### 4.3. Patrol Inspection

The requests for the inspection process come from inspectors and operators who need to ensure machine running status. It is beneficial to detect problems in the early stage to reduce the costs of maintenance by a large margin. From IoT service, sensor data are transferred to the IoT server and checked by preset boundaries. However, the running problem is sometimes neglected by merely checking the sensor data, which need to be inspected by inspectors near the running machine.

As demonstrated in Figure 3, by our system, each running machine can be associated with a unique QR code, which is further related to the ID in the IoT server. After establishing the binding, the inspector can acquire running data from the IoT server in real-time. Combined with the actual machine running status, it is more accurate to detect running problems and then fix them. After inspection, the inspector can upload the annotation to explain the noticeable information. When another inspector checks the same machine, he can browse the annotation and modify it.

## 5. User Study

In this section, we conducted a user study to investigate the system usability and difficulty through setting up tasks about the design, sale, and inspection for participants.

### 5.1. Procedure

In our user study, we recruited 20 participants (2 females, 19 to 41 years old, mean =27.7, Std. Dev. =6.58). All participants had over 2 years of experience using PCs, smart phones, or tablets (three participants had tried AR applications before).

At the start, each participant read the study information, answered a questionnaire, and signed the consent form. Then, volunteers distributed a prepared system user guide and task information to participants. After completing the reading, we performed a simple training process and Q & A about our system, covering the functions, explanations, usages, and tasks.

The user study was held in an actual factory, which had cutting machines and a large free space. The whole study consisted of three tasks that covered design, sales, and inspection application, introduced in the previous section. The detailed procedures of the three tasks are illustrated in Table 1. The procedures of the tasks were set at a time limit. When the participant did not complete the procedure within the time limit, it was regarded as a failure in this procedure.

To test the usability and difficulty of the study, we exploited the single ease question (SEQ) [44] to measure task difficulty, the subjective mental effort questionnaire (SMEQ) [45] to test the level of mental effort needed during the task, and the system usability scale (SUS) [46] to estimate usability.

### 5.2. Results

Table 2 reports the mean completion time and failure number of each step. On average, participants spent 12.6 min in the design task, 9.9 min in the sales, and 13.6 min in the inspection. Furthermore, participants only made a small number of errors. Among all participants, twelve persons did not make any errors. In all steps, process 2.3 took the most time and had the highest failure number. The reason is that it was the first time for most participants to operate the virtual object in a real environment. Moreover, operating the virtual model in 3D space with a 2D multi-touch screen also increased the task difficulty.

**Figure 6:**
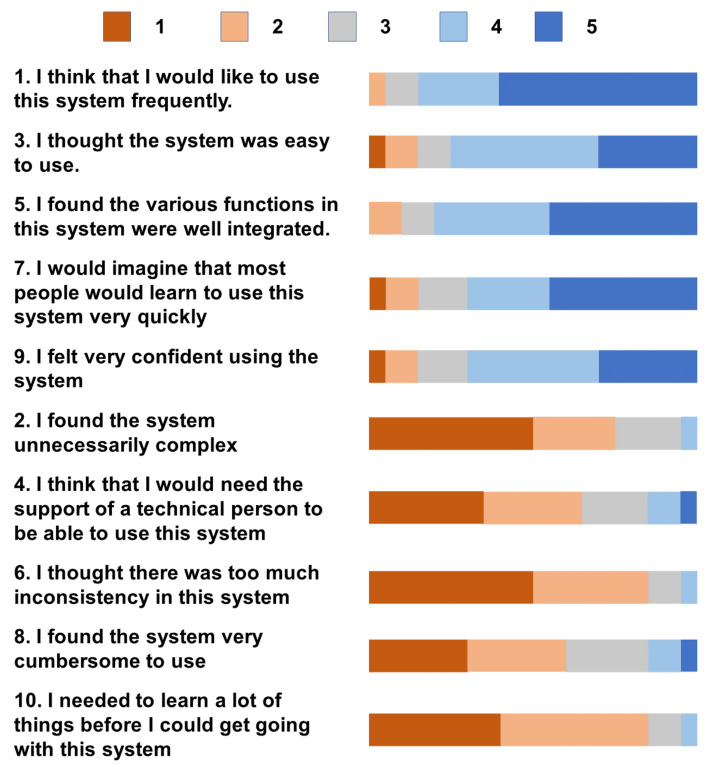
Detailed results for each question in SUS [46] (1: strongly disagree; 5: strongly agree).

The detailed SUS [46] results are presented in Figure 6. The mean score of all participants was 75.4 with IQR [71.5,86.3]. From the SUS results, it is obvious that most participants had good user experiences using MUCStudio and MUCView to complete the tasks. For most participants, it was their first time using the AR system. The usability of our system is appreciable.

Regarding task difficulty, the median value of SEQ was 6, with IQR [5–7] (SEQ 1: very difficult; 7: very easy—the higher, the easier), while the median result of SMEQ was 12 with IQR [8–15] (0: not hard at all–150). It is obvious that both SEQ and SMEQ performances show that completing the tasks were not hard. In particular, all participants received a simple training process before completion, and most had not used an AR system before. In actual application, after systemic training, users could have better performance.

## 6. Conclusions

In this paper, focusing on industrial applications, we proposed a multi-user collaborative AR system composed of MUCStudio for content generation, MUCView for providing AR experiences, and MUCServer for database and collaborative services. On the one hand, the system could be applied to various industrial stages that contain designs, sales, inspections, etc. On the other hand, three collaborative ways are presented among the different roles, covering remote support, collaborative editing, and annotation. To achieve collaborative localization and improve user experience, we implemented the algorithms of local map establishment, global map registration, optimization, and network synchronization. Based on the proposed system, we develop three laser cutting machine applications, which include the cutting head design, production line sale, and patrol inspection of a cutting machine. Furthermore, to evaluate the system, we conducted a user study that covered three tasks through SEQ, SMEQ, and SUS, demonstrating use difficulty and user experience.

## Figures and Tables

**Table 1:** Detailed procedures of experimental tasks. The unit of the time limit is “minute”.

Task ID	Description	Time Limit
**1. Design**		
1.1	Download the specific CAD model and transform it to obj format in MUCStudio.	4
1.2	Load both cutting machine and head models.	2
1.3	Add the marker and set the pose of marker, cutting machine, and head.	8
1.4	Save the scene and export it to MUCView.	2
1.5	Print the marker and paste it to the designed position.	3
1.6	Run MUCView and load the scene file.	2
1.7	Scan the marker to display the scene model.	2
**2. Sale**		
2.1	Run MUCView and load the initial model and layout of production line.	5
2.2	Choose the appropriate position to display the production line.	2
2.3	Adjust the position and orientation of the production line to the given target layout.	10
2.4	Save the scene.	2
**3. Inspection**		
3.1	Generate a QR code and bind it to the given machine ID.	2
3.2	Print the QR code and paste it to the cutting machine.	3
3.3	Scan the QR code to display the running information.	2
3.4	Add a slice of annotation and upload it to the MUCServer.	5
3.5	Restart the MUCView and load the annotation information.	5
3.6	Modify the annotation and re-upload it.	5

**Table 2:** Completion time and failure number of each experimental process. The unit time of completion time is “minute”.

Task ID	Completion Time/ Failure Number	Task ID	Completion Time/ Failure Number	Task ID	Completion Time/ Failure Number
**1. Design**					
**1.1**	2.2 min/0	**1.2**	1.1 min/0	**1.3**	4.7 min/2
**1.4**	0.9 min/1	**1.5**	2.1 min/1	**1.6**	0.8 min/0
**1.7**	0.8 min/0				
Total		12.6 min/4			
**2. Sale**					
**2.1**	2.9 min/0	**2.2**	0.8 min/0	**2.3**	5.7 min/3
**2.4**	0.5 min/0				
Total		9.9 min/3			
**3. Inspection**					
**3.1**	1.5 min/0	**3.2**	1.9 min/0	**3.3**	0.9 min/0
**3.4**	3.1 min/1	**3.5**	2.8 min/0	**3.6**	3.4 min/1
Total		13.6 min/2			

## Data Availability

The data presented in this study are available on request from the corresponding author.
